# The Increased Risk of Joint Venture Promotes Social Cooperation

**DOI:** 10.1371/journal.pone.0063801

**Published:** 2013-06-04

**Authors:** Te Wu, Feng Fu, Yanling Zhang, Long Wang

**Affiliations:** 1 Center for Systems and Control, State Key Laboratory for Turbulence and Complex Systems, College of Engineering, Peking University, Beijing, China; 2 Program for Evolutionary Dynamics, Harvard University, Cambridge, Massachusetts, United States of America; 3 Institute of Integrative Biology, ETH Zurich, Zurich, Switzerland; University of Maribor, Slovenia

## Abstract

The joint venture of many members is common both in animal world and human society. In these public enterprizes, highly cooperative groups are more likely to while low cooperative groups are still possible but not probable to succeed. Existent literature mostly focuses on the traditional public goods game, in which cooperators create public wealth unconditionally and benefit all group members unbiasedly. We here institute a model addressing this public goods dilemma with incorporating the public resource foraging failure risk. Risk-averse individuals tend to lead a autarkic life, while risk-preferential ones tend to participate in the risky public goods game. For participants, group's success relies on its cooperativeness, with increasing contribution leading to increasing success likelihood. We introduce a function with one tunable parameter to describe the risk removal pattern and study in detail three representative classes. Analytical results show that the widely replicated population dynamics of cyclical dominance of loner, cooperator and defector disappear, while most of the time loners act as savors while eventually they also disappear. Depending on the way that group's success relies on its cooperativeness, either cooperators pervade the entire population or they coexist with defectors. Even in the later case, cooperators still hold salient superiority in number as some defectors also survive by parasitizing. The harder the joint venture succeeds, the higher level of cooperation once cooperators can win the evolutionary race. Our work may enrich the literature concerning the risky public goods games.

## Introduction

Grouping of individuals, no matter what mechanism leads to such groupings, plays a decisive role in the survival of group members, notably under austere conditions. Collective action of individuals incurs a cost to themselves and provides a common benefit to community members, even to those who have not contributed. Such cooperative behaviors are widespread in the real world [1]–[4], [6], [12], [23], [24], [26], [27], [32], [33], [36], [37], [40], [46], [51]. Prominent examples include the food-sharing system in some African tribes, alarm calls in meerkats, and stalk-shaped forming in amoeba, just to name a few [5], [7], [9], [10], [22]. A social dilemma arises when someone in the group withholds its contribution and instead free rides on others' efforts. In this case, the best choice for a group and that for an individual are at odds. The temptation of free-riding thus threatens and further breaks down social cooperation. However, cooperation is almost always viable and robust in the natural world. To under the conundrum of cooperation, a number of mechanisms have been proposed over the last decades. For an excellent review, please see Ref. [29].

Risk is ubiquitously involved in many collective actions [14]–[16]. In these situations, members can benefit from the public pool resource only if the risk of failure is smoothly avoided. The avoidance of the risk hinges significantly on the cooperativeness of the group [8], [21], [25], [28], [31]. The risk perception of people might have been quite important for primeval humans living in small communities and mainly feeding on joint hunting. In this scenario, large preys such as wild boar can easily run away if hunters mostly engage in the rounding up halfheartedly, and other few hunters' effort flows away in vain. In contrast, if most participating hunters contribute to the hunting labor, there is a large chance of reaching the collective goal of capturing the predator, and thus all members enjoy the fruit of the collective effort [39], [41]. In recent years, there has been increasing recognition of the need to address issues surrounding risk prevention in view of frequent outbreaks of global financial crisis. Risk comes up with investing in stock market and banking ecosystems [11]. To produce the economic benefit in these systems, they must be collectively high cooperative. Small dysfunction may lead to unwanted breakdown in a cascading way. More recently, Ref [28] has pointed out that the global climate change constitutes a typical example of the tragedy of the commons, where the occurrence of the risk plays a key part in guiding individual's decision making in donating. The experimental findings show that high risk rate indeed induces higher donation of individuals into the climate account to avoid the loss of their private savings. Inspired by this experiment design, Wang et al. [21] have theoretically investigated how altruistic donators fare in the threshold public goods game, where individuals donate so as to achieve the collective target to prevent their remaining money from being lost rather than to obtain enhanced benefit of their investments.

Here, we model the joint venture with the risk public goods game [13], [30], [35], [39], [41], [47], [48]. In the joint enterprises involving risk, the production and subsequently the allocation of the public wealth depends not only on the presence of each cooperator but also crucially on how cooperative the group is. Or rather, in order to succeeding in receiving the public goods, all group members must surmount failure risk firstly. The more number of players willing to cooperate, the larger likelihood they succeed. Our model suggest that aggregate investment of cooperators has two bearings. Firstly, it is used to resist failure. Once succeeding, it serves as the base of enhanced benefit on the other hand. Obviously the former is the prerequisite for the later. This stands in stark contrast with the traditional settings where cooperators each, if present in the group, would bring equal benefit unconditionally to all interactive members [17]–[19], [34], [38], [42], [45]. Furthermore, although the joint venture, if successful, can be beneficial to the group, the fear of failure may coerce some individuals turning to voluntary participation [43], [44], [49], [50], since both animals and humankind possess the instinct of circumventing risk. That is to say, risk-averse individuals tend to abstain away from the risky public goods game and rely on some autarkic way of life, whereas the risk-preferential ones tend to engage in the risky public goods game in hoping of acquiring high return. Thus a natural third strategy, say loner [18], [19], emerges along with cooperation and defection. Joint hunting can be costly and risky but can benefit the community, whereas the growing mushrooms (i.e., loner) is risk averse. We focus on under what risk pattern cooperation can get established, and under what other conditions loner can evolve to prevail.

At the center of this problem is how likely risk occurs given the cooperativity of the focal group (which we can call risk function). Instead of using the step function, here we consider three representative types of risk function. Most interesting is the sigmoid function, which embodies the philosophy that less than required amount of cooperation is hardly better than none [28], [31], yet still ensures the success of the joint venture with a small likelihood. Once the amount arrives at some critical point, the probability that the group produces the public goods is quite optimistic, and additional increment in contribution values less and less [5], [20], [28], [31]. Collectively hunting large scale prey in the African wilderness may be an intimately related analog. Contrasting with the traditional public goods game [18], [19], [45], risk is a double-edged sword in such case. On the one hand, groups of low cooperativity can hardly accumulate the effort required to avoid failure. Members evolutionarily pursue noncooperative option. Risk in this situation leads to rapid group-breakup. On the other hand, a successful group is necessarily of high cooperation. Cooperators average higher return, raising the likelihood of, if not winning, at least coexisting with defectors. Furthermore, our findings suggest that the option to abstain from the risk public goods game avoids the impasse in states of all defection. To this evolutionarily stable equilibrium, loners undertake the role of alleviating the risk, since the abundance directly determines the composition of the actual interacting group size. Ironically, although the loner strategy extricates the system from navigating towards the pure state of all defectors, they are eventually engulfed by the risk-loving participants. Compared with public goods game free of risk, the stabilized cooperation sees great improvement, which in turn helps the free riders so much for them also able to outperform the loners.

### A minimal model

Consider a well-mixed population of infinite size. Each individual adopts one of the three strategies, Cooperation (*C*), Defection (*D*) and Loner (*L*). Denote by 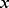, 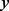 and 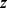 the fraction of *C*s, *D*s and *L*s, respectively. The normalization condition ensures 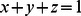. The population is subject to natural selection. Individuals accumulate payoffs by either participating in the risk public goods games, or feeding on some autarkic way of life [18]. For a group composed of 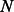 individuals randomly sampled from the population, those individuals (C+D) except L play the risk public goods game: cooperators contribute some fixed amount 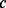, whereas defectors nothing. The success of the group crucially depends on its cooperativeness. The dependent relationship is characterized by a risk function 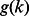 with 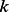 as the number of cooperators. Only successful group can distribute the return of the public good. At this time, the net payoff for cooperators and defectors is given by 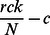 and 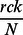 separately, where 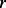 is the enhancement factor. Therefore, the payoff for cooperators and defectors is expected to be 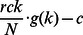 and 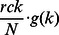. Once the group fails to avert the risk, the contribution of cooperators produces nothing, and defectors get no benefit and incur no loss. The risk-averse individuals (Loners) obtain a constant income 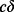. For simplicity we set 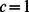 throughout.

We here describe the population dynamics by the replicator equations, where a strategy's payoff determines the growth rate of its abundance within the population:
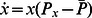


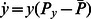


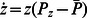
where 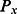, 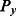 and 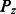 are the net payoffs for strategy 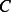, 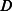 and 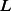, and 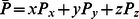 is the average payoff in the population. In infinite population, this approach depicts deterministic selection since the abundance of a strategy increases at a rate given by the difference between the payoff of this strategy and the average payoff of the population.

The calculation of the average payoff to each strategy can be accomplished by implementing the following procedure. From time to time, a focal individual (cooperator or defector) combining other 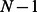 other randomly chosen individuals constitutes an interaction group. Owing to the infiniteness of the population, the random sampling implies that the composition of these 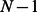 individuals follows a binomial distribution. Individuals's strategies are pre-assigned and do not change with respect to the group formation. In fact, the probability that there are 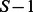 participators among the 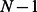 individuals can be calculated as 
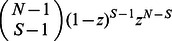
, where 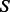 can take the positive integer from 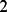 to 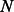. Of the 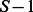 participators, the probability that there are 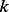 cooperators and the rest defectors is 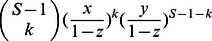. In such a group, the expected payoff is 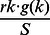 for a focal defector, and 
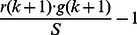
 for a focal cooperator. Averaging this quantity over all possible configurations of the group, we can foretell the payoff of a defector and a cooperator, respectively, as 
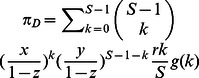
 and 
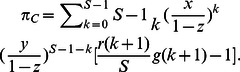
 Thus,
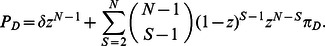



and
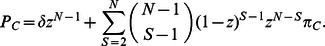



The first term in the right-hand side of 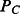 denotes that one cooperator is coerced to act like a loner since all other 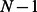 selected individuals are loners.

We have introduced function 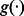 to characterize the probability with which a group avoids the failure risk successively. For self-evident reasons, these two fundamental conditions, 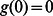 and 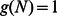, should be satisfied. Meanwhile, 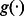 should be non-decreasing in term of the cooperation level of a group in order to make it have biological implications. Such functions are numerous. For the sake of generality, we set 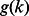 to be
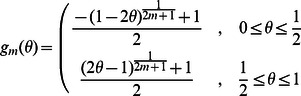
with 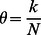 being the cooperativeness of the group given that there are 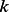 cooperators among the 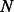 actual participators. By regulating 
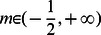
 the function 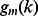 exhibits very rich shapes, which can represent various patterns concerning the dependence of the group's success on its cooperativeness. For example, in the case of 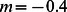, 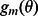 is very closely to 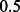 in a wide range of 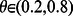, meaning that the requirement for the success of the group is demanding. 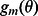 is a linear function for 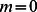, indicating that the contribution of every cooperation weighs equally in averting the risk. The condition 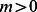 renders 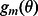 to be a typical sigmoidal function. Of interest is that we can derive the step function with 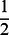 as the jump point as 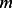 approaches 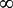. We here would like to pick up these three representative 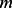
s, say inverse-sigmoidally cooperativeness-dependent pattern (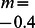), linear cooperativeness-dependent pattern (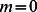), and sigmoidally cooperativeness-dependent pattern (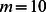) to probe how they would influence the evolutionary competition of the three strategies.

## Results

### Population dynamics when loners are absent

**Figure 1:**
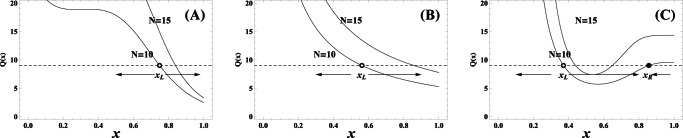
Population dynamics whenever only cooperators and defectors compete to survive. The intersection of 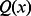 with horizontal line 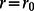 (dashed line) represents the values of fraction of cooperators 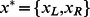 (if 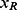 exists) at which payoff of cooperators is equivalent to that of defectors, i.e., 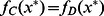. 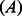 Group's success is inverse-sigmoidally dependent on the cooperativeness of the group. 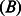 Group's success is linearly dependent on the cooperativeness of the group. In these two cases, scenarios with none, a unique interior fixed point are possible as 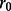 changes. 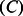 Group's success is sigmoidally dependent on the cooperativeness of the group. Intriguingly, for this pattern, the population dynamics exhibit very rich dynamics: scenarios with none, one and two interior fixed point are possible as 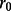 changes. Parameters 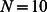 and 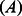 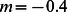, 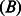 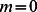, and 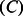 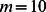.

Before entering into the full model admitting all the three strategies, we first consider the special cases where the Loner strategy is absent (i.e., 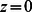). In the absence of the loners, the replicator equation 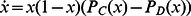 suffices to character the population dynamics where 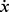 means the time evolution of the abundance of cooperators. Cooperators and defectors compete to survive. The group size 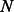 of interacting individuals remains unchanged over time. The payoff difference of cooperators and defectors reads 
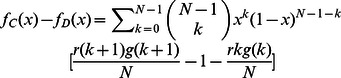
. By setting 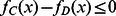, we can obtain 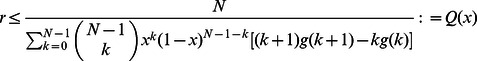, equivalent to the dominance of defectors over cooperators. Whenever the success of the production of common resource linearly relies on the cooperativeness of the group, say 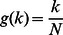, we can accurately derive the mathematical formula of 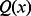 as 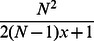 (see Text S1). As for inverse-sigmoidally cooperativeness-dependent and sigmoidal cooperativeness-dependent patterns, there is no possibility to get the simple mathematical expression of 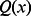. We can nonetheless look into the properties of the algebraic equation 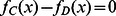 by numerical solving. Figure 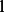 demonstrates that there exists a threshold value 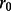 of 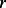 for the three patterns. For 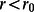, defectors outperform cooperators, driving the population towards the full defective state, irrespective of the initial frequency ratio of the two strategy types. If the reverse of the inequality is true (i.e, 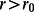), the population dynamics vary with the risk removal pattern. In both cases where the success depends inverse-sigmoidally and linearly on the group's cooperativeness, the system has a unique interior unstable fixed point 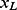 (Figure 1A, 1B), suggesting that the evolutionary fate of cooperators crucially depends on the initial abundance 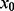 of cooperators. The abundance 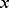 of cooperators evolves to ever lower value if the initial fraction of cooperators 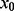 is 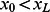 and the population ends up with all defectors, but to ever higher value and cooperation gets stabilized if 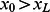.

Very intriguing dynamics emerge for the sigmoidally cooperativeness-dependent pattern. The equation 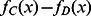 possibly has no, unique, two nontrivial roots as 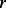 varies, corresponding to the rich dynamics that admits none, unique unstable and, one stable and one unstable interior fixed point (Figure 1B). Of interest is that the sigmoidal risk removal patter leads to the appearance of two mixed internal equilibria whenever 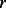 approaches 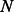 from less than 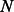, overthrowing the absolute dominance of defectors over cooperators. Cooperators become disadvantageous when rare (below 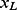) and when abundant (above 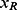) with perfectly distinct underlying causes. For a low cooperative population, groups have fat chance to succeed. Contribution of cooperators vanishes without any return, which hastens the demise of cooperators. As a consequence, the population is eventually absorbed into a full state of all defectors. For a highly cooperative population, collective coordination becomes easier to achieve. Groups' success is almost for sure, which unbiasedly benefits the participants. The aggregate payoff of defectors dotted in these groups exceeds that of cost-bearing cooperators. Defectors therein reproduce at a more faster rate, decreasing the fraction of cooperators in the population. Therefore, the population consisting of above 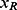 cooperation is tugged back. Once the cooperation level lies between the two internal equilibria, randomly formed groups are still likely to succeed, but not so frequently as when 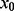 is above 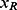. The intermittent success of groups indeed reduces defectors' exploitation on cooperators, resulting higher average payoff of cooperators than defectors. Cooperators increases in abundance. As a result of these two considerations, the coexistence of cooperators and defectors becomes stable with the former accounting a fraction 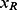 (See Text S2).

Comparing the three panels in Figure 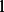 demonstrates that the presence of risk substantially changes the population dynamics. Cooperators have chance to survive provided that their initial fraction is sufficiently high to overcome the coordination barrier (i.e., risk circumventing). However, whether cooperators can pervade into the whole population crucially relies on the way how the group's success depends on its cooperativeness. Actually, we have found that the population dynamics can be generally classified into categories in terms of in terms of the risk avoidance function. Whenever the role of cooperators' contribution weakens slowly (i.e., 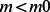) as cooperators increases, there just exists a unique unstable internal fixed point (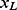 in Figure 1) separating the whole area into two sections, one the attraction basin of defectors and the other that of cooperators. Whenever the role ramps down quickly (i.e, 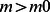), another stable fixed point appears corresponding to the mixed state of cooperators and defectors besides 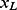.

### Population dynamics when cooperators, defectors and loners compete to survive

**Figure 2:**
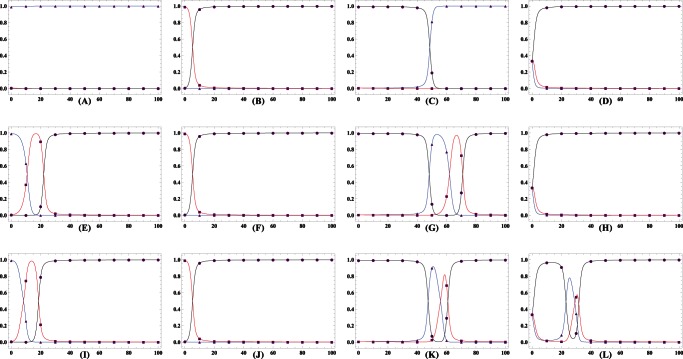
Population dynamics whenever cooperators defectors, and loners compete to survive. The lines embedded with solid triangle, square, and circle represent the evolutionary trajectories of cooperators, defectors and loners, respectively. For 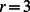, in both the linearly-dependent and sigmoidally-dependent patterns, the evolution share the property that when cooperators abound, it is better to defect, while defectors' prosperity puts the loners in the advantageous place forever, and thus loners take over the whole population. In the inverse sigmoidally-dependent pattern, loners do thrive ensuing the defectors' abundance. An exception emerges while the population consists of most cooperators, it drives to the full cooperative state. Loners win the evolutionary race when the population starts with a state in which cooperators and defectors, and loners have the same share. Parameters 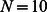, 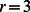. Upper row 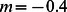, middle row 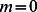, and low row 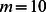.

Let us now focus on what influence the risk-averse loners exercise on the population dynamics. Figure 2 clearly illustrates the evolutionary trajectories of the population starting with four typical mixes under the three different risk removal patterns for 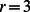. Each mix corresponds to a mixed state of cooperators, defectors and loners. For such small 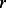, loners pervade the whole population in most of the twelve cases. Of interest is that cooperators are still able to win the evolutionary race provided that the population starts with cooperators themselves or loners holding the absolute majority for 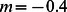. The rationale behind this phenomena can be intuitively comprehended. To flip the coin determines the success of groups consisting of defectors and cooperators who fail to compete with loners from the perspective of statistics. Only when several cooperators agglomerate together, such high cooperative groups with probabilities close to 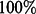 succeed in averting the failure risk. Cooperators from these groups are able to beat loners and defectors. Differently, the likelihood is enhanced for the cases 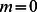 and 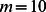 when the group's cooperativeness is above 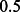 yet below 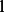. Consequently, interspersed defectors can more frequently exploit and therein outperform the cooperators, which occasions the prevalence of defectors following the spawning of cooperators, as do loners after defectors. Once dominating, loners establish forever, vanishing the cyclical dominance of rock-paper-scissor type.

**Figure 3:**
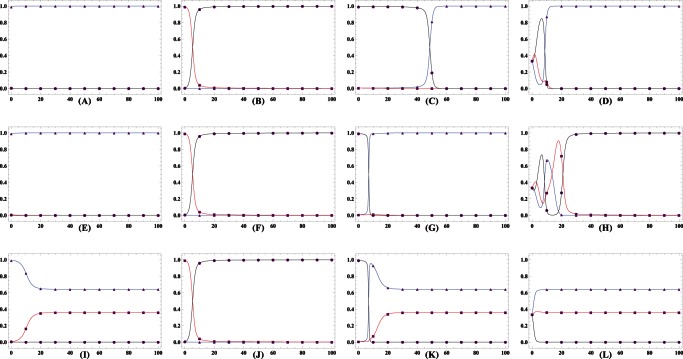
Population dynamics whenever cooperators defectors, and loners compete to survive. The lines embedded with solid triangle, square, and circle represent the evolutionary trajectories of cooperators, defectors and loners, respectively. For 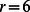, irrespective of the risk removal patterns, loners becomes the unique victor for the population starting with defectors accounting for the absolute majority. The abundance of loners or cooperators invariably leads to the establishment of cooperators in the inverse sigmoidally and linearly dependent patterns, while leads to the coexistence of cooperators and defectors in the sigmoidally-dependent pattern. Starting with the point of 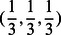 responding to the fraction of cooperators, defectors, and loners, the population navigates to extremely different equilibrium states under the three risk removal patterns. Parameters 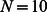, 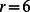. Upper row 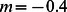, middle row 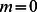, and low row 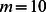.

**Figure 4:**
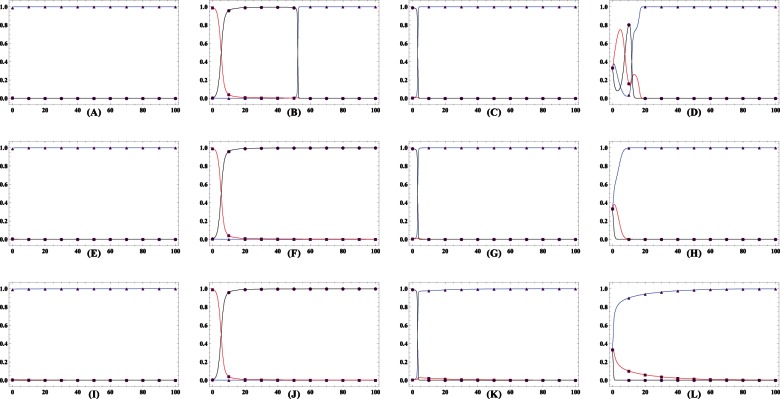
Population dynamics whenever cooperators defectors, and loners compete to survive. The lines embedded with solid triangle, square, and circle represent the evolutionary trajectories of cooperators, defectors and loners, respectively. For 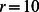, cooperators pervade into the whole population in the inverse sigmoidally and linearly dependent pattern, while loners completely dominate in the sigmoidally pattern, when defectors abound at the starting point. In the other typical cases, cooperators uniformly take over the whole population, though sometimes the evolutionary processes are different. Parameters 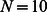, 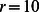. Upper row 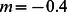, middle row 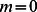, and low row 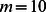.

As 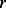 rises to the moderate level, the cycle still does not exist, while cooperators have more chances to establish (Figure 3). Irrespective of the risk removal pattern, loners take over the whole population if defectors account for the overwhelming majority at the outset of the evolution (Figure 3B, 3F, 3J). Depending on the risk removal patterns, the population exhibits rich dynamics. Of the remaining three starting mixes, cooperators and defectors always coexist under the sigmoidal pattern (Figure 3I, 3K, 3L) while cooperators evolve to be the only survivors in the inverse sigmoidally pattern (Figure 3A, 3C, 3D). However, loners win out after a transiently cyclical dominance for the population starting with equal fractions under the linear pattern (Figure 3H). This also implies that the fixed point 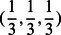 is unstable. The population starting from this point would oscillate around it with the amplitude increasing. Until eventually, loners homogenize the population. With 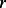 arriving at 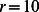, cooperation stands conspicuously advantageous as the evolution most of the time ends up with the triumph of cooperators (Figure 4), apart from that loners occasionally directly spread into the whole population after beating the prevalent defectors in the sigmoidal pattern (Figure 4F, 4J).

**Figure 5:**
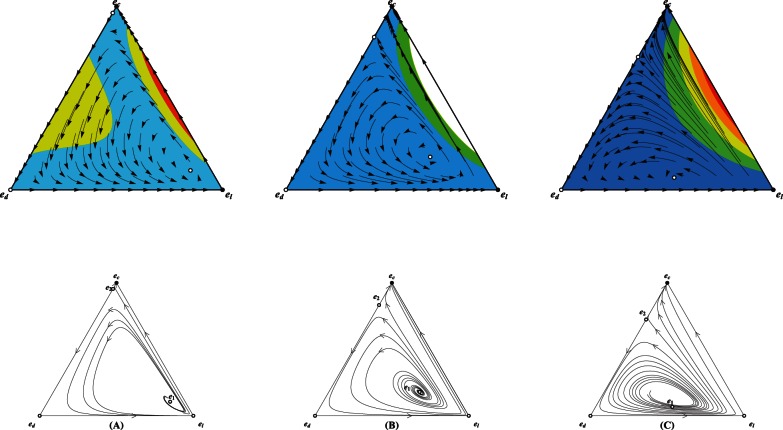
Triangle plots illustrating the population dynamics for defectors (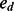), cooperators (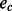) and loners (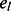) for trajectories starting from all possible initial frequencies for the inverse-sigmoidal risk removal pattern. Each vertex represents a homogeneous population of that pure strategy. 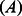 For small interest rate 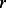, except for the unique nontrivial fixed point (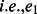) located inside the simplex 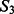, there also exists another nontrivial fixed point located in the line 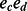 (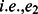). In consequence, the inside area of the simplex 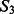 is divided into two attraction basins, with one being loners' and the other cooperators'. 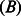 For modest 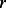, 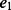 and 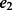 as in plot 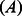 still exist. Instead the cooperators' attraction basin covers absolutely large fraction of the inside area of the simplex 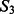, and loners win the evolution for population starting from the remaining area. If defectors are abundant, the population converges to the full cooperative state in a spiral way around the unstable fixed point 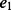. Otherwise, the population directly drives towards 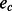. 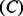 Further increase in 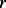 continue to expand the cooperators' attraction basin. It should be noted that even 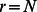, the attraction basin albeit narrow does not vanish. Relevant parameters 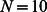, 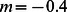 and 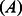 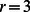, 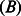 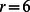, 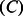 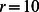.

**Figure 6:**
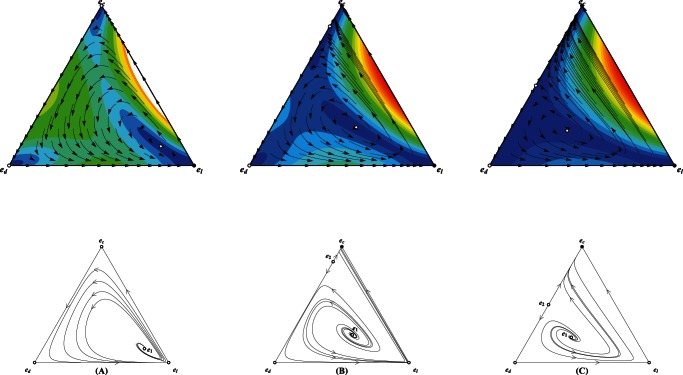
Triangle plots illustrating the population dynamics for defectors (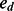), cooperators (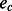) and loners (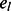) for trajectories starting from all possible initial frequencies for the linear risk removal pattern. Each vertex represents a homogeneous population of that pure strategy. 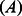 For small interest rate 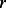, there is only one nontrivial fixed point (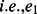) located inside the simplex 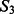. All trajectories starting from inside of the simplex 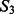 invariable lead to the unique convergent equilibrium 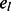. 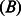 For modest 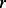, except 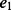, there also exists another nontrivial fixed point located in the line 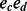 (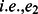). Instead the cooperators' attraction basin covers absolutely large fraction of the inside area of the simplex 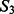, and loners' attraction basin is almost negligible. If defectors are abundant, the population dynamics oscillate around the unique unstable interior fixed point 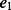 with increasing amplitude and eventually converges to the full cooperative state 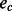. Otherwise, the population directly drives towards 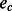. 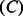 Further increase in 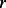 continue to expand the cooperators' attraction basin. It should be noted that even 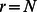, loners' attraction basin, albeit narrows, does not vanish. Relevant parameters 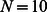, 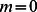 and 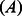 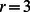, 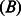 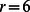, 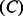 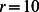.

**Figure 7:**
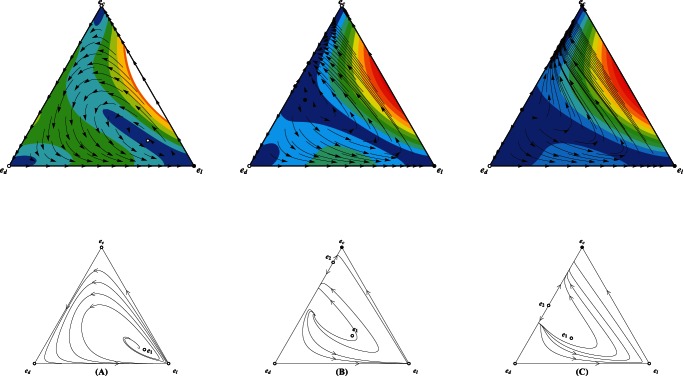
Triangle plots illustrating the population dynamics for defectors (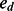), cooperators (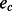) and loners (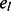) for trajectories starting from all possible initial frequencies for the inverse-sigmoidal risk removal pattern. Each vertex represents a homogeneous population of that pure strategy. 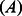 For small interest rate 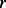, all orbits starting from the inside area of 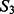 converge to 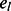. 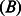 Moderate 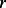 delimits the inside area into two attraction basins. The equilibrium 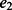, a coexistence state of cooperators and defectors, accounts a relatively large area as its attraction basin. The rest of the inside area is the loner's attraction basin. 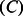 Further increase in 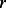 makes the 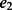 coincide with 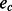 and its attraction area spreads almost the whole inside area. Relevant parameters 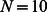, 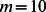 and 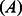 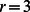, 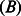 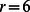, 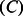 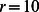.

We now full characterize the population dynamics by a three dimensional simplex 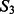, whose each point has three components denoting the fractions of cooperators, defectors, and loners respectively. For 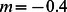, a small enhancement factor 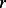 divides the inside of the simplex 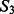 into two attraction areas, with the larger one being the loners' and the other one cooperators' (Figure 5A). In both linearly and sigmoidally cooperativeness-dependent patterns, the same enhancement factor 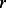 leads to that loners progressively pervade the entire population and eventually dominate, reflecting by that each orbit starting from any point inside 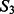, with exception of fixed points if they do exist, drives towards and ends at the pure state of loners (Figure 6A, 7A), resembling the results widely reported in the public enterprizes absent of risk. This distinction can be attributed to the following interpretations. As have established, loner is the payoff-maximizing strategy for small 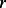 in the absence of the failure risk. The presence of risk indeed reduces the expected payoff of both defectors and cooperators in comparison to most traditional studies (i.e., without risk) for identical group composition. Payoff of loners remains unaffected. Though depending on the cooperativeness, the group's success is not so hard in the linear and sigmoidal patterns as that in the inverse-sigmoidal pattern, therefore defectors parasited in groups of most cooperators and loners have much more chances to hitch cooperators' contribution. The exploitation induces the evolutionary trajectory to move forward along the direction of increasing defectors, who are emulated by the loners. Differently, as the group's success is such strongly on its cooperativeness for 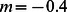, the successful group must bring much payoff for cooperators than for defectors, thus the trajectory starting in the area covering immensely few defectors would drive towards the full cooperative state.

We are most concerned about whether cooperation can survive whenever 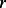 approaches yet sill below 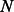. It is shown that the presence of risk extinguishes the population dynamics of rock-paper-scissors type. Whenever the success strongly depends on group's success (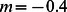 and 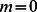), the attraction basin of cooperators covers a relatively large area of the inside of the simplex 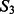 (Figure 5B, 6B). For the evolution starting from the point near the line 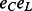, it directly progresses to the full cooperative state. Of interest is that whenever the starting point is located in the remaining part of cooperator' attraction area, it takes a different way to arrive at 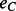. The dynamics of trajectory exhibit oscillatory behavior. Owing to the instability of 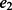, the multitude of the oscillation keeps growing. Before arriving at the attractors, if defection can rise to a high level, defectors in turn are defeated and vanish, since the required threshold of cooperators for groups' success is hard to satisfy. In this situation, loners play the role of salvaging the population out of the conundrum of all defecting. Once the abundance of these saviors suffices to substantially regulate the actual interaction group size, cooperators are able to swiftly soar and eventually homogenize the whole population. Therefore, the increment of defectors are self-destructive, and loners bridge the population to the full cooperative state after transiently dominating. An exception is that if defectors are almost pervaded into the whole population, loners would take over before cooperators procure chance to deluge. For 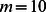, group's success depends on its cooperativeness to the weakest extent of the three invested patterns. The oscillatory trajectory around 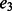 quickly converges to the coexistence state of cooperators and defectors. The attraction basin of this attractor covers substantial fraction of the simplex 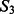 (Figure 7B). For 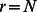, all interior fixed points disappear. As have shown, cooperators and defectors show positive frequency dependence whenever loners are absent. However, stability of defection is eliminated with the addition of loners while stability of cooperation is robust. Although the microscopic roadway differs for different risk removal pattern, the system mostly converges to the full cooperative state (Figure 5C, 6C, 7C). It should be noted that the attraction area of loners, albeit stingily narrow, still exists, which differs from the situation without risk.

We would like to make longitudinal comparison with relevant works. We extend Pacheco *et al* [31] model, which is just the case of 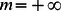 in our model, to more general cases. Generally speaking, the effect of the risk removal pattern can be categorized into two classifications. There exists a threshold 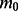 of 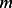 (see Table S1). For 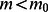, defectors and cooperators display the property of positive frequency dependence. Initial staring point determines their evolutionary fate. For 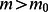, the population dynamics can possibly admit none, unique, and two equilibria as 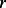 varies. Therefore, we have studied two very typical cases 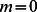 and 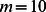, since they suffice to address the population dynamics. To better compare the results, especially when loners are introduced we have also studied a third case of 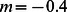. The most interesting regime is the emergence of two equilibria (i.e., 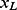 and 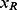 in Figure 1C). And 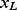 corresponds to the collective coordination barrier. Once overcome, the population is eventually stabilized at 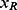, defining the final fraction of cooperators. Actually, presence of risk plays diametrically opposite role in sustaining cooperation. For population consisting of rare cooperators, risk makes cooperators' effort almost drain away, and thus speeds up the doom of the population. If initially cooperators abound, they are always able to coexist with defectors. These works [21], [35] also dealt with risk in public goods game, but altruists are willing to cooperate just in order to protect remaining account from losing, which is decisively different from our model. Even so, risk also leads to the emergence of two equilibria possessing similar property of stability as in our model. This result was experimentally verified in [28]. Therefore, risk universally plays the role of double-edged sword in public goods transactions. For more realistic situation, individuals are instinctively inclined to abstain from engaging in the risk public goods enterprizes but subsist on some safe solitary actions. For moderate 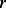, loners hold a very small area as their eventual territory. But most time, they just act to extricate the population especially whenever defectors are abundant. These results may be conducive to the risk management.

## Discussion

We have extended the traditional public goods game to the systems in which groups of interacting individuals would undergo the foraging failure risk. Overcoming the risk is positively dependent on group's cooperativeness. Only those groups circumventing the risk successfully can create the public resource and subsequently divide evenly to all engaging individuals. The dependency is characterized by the function 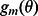. Three strategies, loner, cooperation and defection are feasible to individuals. For very low interest rate, risk is detrimental to the evolution of cooperation most of the time. However, once cooperators enjoy the absolute dominance in number, they are able to take over the whole population. As 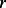 increases to moderate level, three risk removal patterns induce qualitatively different dynamics. If the likelihood that the success of the joint venture relies on the group's cooperativeness in a linear or more strong way, defectors fail to pervade the whole population. The population is always stabilized at a pure state of either all cooperators or all loners, with 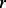 determining their respective attraction basins. Invoking the sigmoidal function to represent the risk function embodies the idea that “three in a boat, two row”. The third one can always enjoy the efforts of the two rowers. In this setting, the dependency is not so strong as in the linear pattern. Results show that the basin of attraction of loners accounts for a nonnegligible fraction of the whole state space. Most frequently defectors can coexist with cooperators by parasitizing on cooperators. Taken together, the harder the group succeeds, the higher cooperation level the population arrives provided cooperators can build up. Increasing 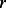 strengthens the role of loners acting as the rescuers than as the terminators of the population.

Our model captures the risk elements in the collective coordination, making population dynamics differ essentially from the traditional public goods game in two fronts. On the one hand, for the population just consisting of cooperators and defectors, when cooperators are abundant, they will eliminate, or at least coexist with defectors. The positive frequency dependency of cooperators relaxes the dilemma that defectors always wipe out cooperators especially for the modest enhancement factors. On the other hand, loners most of the time play the role of salvaging the population out of “the tragedy of the commons” [13], and help the population drive towards the full cooperative state, which is contrast with the cyclical dominance of cooperators, defectors and loners widely reported in previous studies [17]–[19].

## Supporting Information

Text S1 (TEX)

Text S2 (TEX)

Table S1 (TEX)

## References

[1] Axelrod R (1984) The Evolution of Cooperation. New York: Basic Books.

[2] Axelrod R, Hamilton WD (1981) The evolution of cooperation. Science 211: 1390–1396. 7466396 10.1126/science.7466396

[3] Binmore KG (1994) Playing Fair: Game Theory and the Social Contract. Cambridge: MIT Press.

[4] Boyd R, Richerson PJ (1985) Culture and the evolutionary process. Chicago, IL: University of Chicago Press.

[5] Boyd R, Mathew S (2007) A narrow road to cooperation. Science 316: 1858–1859. 17600207 10.1126/science.1144339

[6] Brandt B, Hauert C, Sigmund K (2003) Punishment and reputation in spatial public goods games. Proc. R. Soc. B 270: 1099–1104. 10.1098/rspb.2003.2336 PMC1691345 12803901

[7] Fehr E, Gächter S (2002) Altruistic punishment in humans. Nature 415: 137–140. 11805825 10.1038/415137a

[8] Feng F, Rosenbloom DI, Long W, Nowak MA (2010) Imitation dynamics of vaccination behaviour on social networks. Proc. R. Soc. B 278: 42–49. 10.1098/rspb.2010.1107 PMC2992723 20667876

[9] Gürerk Ö, Irlenbusch B, Rockenbach B (2006) The competitive advantage of sanctioning institu-tions. Science 312: 108–111. 16601192 10.1126/science.1123633

[10] Gurven M (2004) Reciprocal altruism and food sharing decisions among hiwi and ache hunter-gatherers. Behav. Ecol. Sociobiol. 56: 366–380.

[11] Haldane AG, May RM (2011) Systemic risk in banking ecosystems. Nature 469: 351–355. 21248842 10.1038/nature09659

[12] Hamilton WD (1963) The evolution of altruistic behaviour. Am. Nat. 97: 354–356.

[13] Hardin G (1968) The tragedy of the commons. Science 13: 1243–1248. 5699198

[14] Chen XJ, Szolnoki A, Perc M (2012) Averting group failures in collective-risk social dilemmas. EPL 99: 68003.

[15] Chen XJ, Szolnoki A, Perc M (2012) Risk-driven migration and the collective-risk social dilemma. Phy. Rev. E 96: 036101. 10.1103/PhysRevE.86.036101 23030974

[16] Chakra MA, Traulsen A (2012) Evolutionary dynamics of strategic behavior in a collective-risk dilemma. PLoS Comput. Biol. 8: e1002652. 10.1371/journal.pcbi.1002652 PMC3426567 22927807

[17] Hauert C, Holmes M (2006) DoebeliM (2006) Evolutionary games and population dynamics: maintenance of cooperaion in public goods games. Proc. R. Soc. B 273: 2565–2570. 10.1098/rspb.2006.3600 PMC1634915 16959650

[18] Hauert C, Monte SD, Hofbauer J, Sigmund K (2002) Volunteering as red queen mechanism for cooperation in public goods games. Science 296: 1129–1132. 12004134 10.1126/science.1070582

[19] Hauert C, Monte SD, Hofbauer J, Sigmund K (2002) Replicator dynamics for optional public goods games. J. theor. Biol. 218: 187–194. 10.1006/jtbi.2002.3067 12381291

[20] Henrich J, McElreath R (2002) Are peasants risk-averse decision makers. Curr. Anthrop. 43: 172–181.

[21] Wang J, Fu F, Wu T, Wang L (2009) Emergence of social cooperation in threshold public goods games with collective risk. Phys. Rev. E 80: 016101. 10.1103/PhysRevE.80.016101 19658768

[22] Kagel JH, Roth AE (1995) The handbook of experimental economics. Princeton: Princeton Uni-versity Press.

[23] May RM (1981) The evolution of cooperation. Nature 292: 291–292. 7254327 10.1038/292291a0

[24] Milinski M (1987) Tit for tat in sticklebacks and the evolution of cooperation. Nature 325: 433–435. 3808044 10.1038/325433a0

[25] Milinski M, Semmann D, Krambeck HJ, Marotzke J (2006) Stabilizing the earth's climate is not a losting game: supporting evidence from public goods experiment. Proc. Natl. Acad. Sci. USA 103: 3994–3998. 10.1073/pnas.0504902103 PMC1449634 16537474

[26] Szolnoki A, Szabó G, Perc M (2011) Phase diagrams for the spatial public goods game with pool punishment. Phys Rev E 83: 036101. 10.1103/PhysRevE.83.036101 21517552

[27] Helbing D, Szolnoki A, Perc M, Szabó G (2010) Evolutionary establishment of moral and double moral standards through spatial interactions. PLoS Comput Biol 6(4): e1000758. 20454464 10.1371/journal.pcbi.1000758 PMC2861625

[28] Milinski M, Sommerfeld RD, Krambeck HJ, Reed FA, Marotzke J (2008) The collective-risk social dilemma and the prevention of simulated dangerous climate change. Proc. Natl. Acad. Sci. USA 105: 2291–2294. 10.1073/pnas.0709546105 PMC2268129 18287081

[29] Nowak MA (2006) Five rules for the evolution of cooperaion. Science 314: 1560–1563. 17158317 10.1126/science.1133755 PMC3279745

[30] Ostrom E (1999) Coping with tragedies of the commons. Annu. Rev. Polit. Sci. 2: 493–535.

[31] Pacheco JM, Santos FC, Souza MO, Skyrms B (2008) Evolutionary dynamics of collective action in N-person stag hunt dilemmas. Proc. R. Soc. B 276: 315–321. 10.1098/rspb.2008.1126 PMC2674356 18812288

[32] Helbing D, Szolnoki A, Perc M, Szabó G (2010) Defector-accelerated cooperativeness and punish-ment in public goods games with mutations. Phys Rev E 81: 057104. 10.1103/PhysRevE.81.057104 20866359

[33] Szolnoki A, Perc M, Szabó G (2009) Topology-independent impact of noise on cooperation in spatial public goods games. Phys Rev E 80: 056109. 10.1103/PhysRevE.80.056109 20365045

[34] Santos FC, Santos MD, Pacheco JM (2008) Social diversity promotes the emergence of cooperation in public goods games. Nature 454: 213–217. 18615084 10.1038/nature06940

[35] Santos FC, Pacheco JM (2011) Risk of collective failure provides an escape from the tragedy of the commons. Proc. Natl. Acad. Sci. USA 108: 10421–10425. 10.1073/pnas.1015648108 PMC3127907 21659631

[36] Perc M (2009) Evolution of cooperation on scale-free networks subject to error and attack. New Journal of Physics 11: 033027.

[37] Perc M, Szolnoki A (2008) Social diversity and promotion of cooperation in the spatial prisoner's dilemma game. Phys Rev E 77: 011904. 10.1103/PhysRevE.77.011904 18351873

[38] Semmann D, Krambeck HJ, Milinski M (2003) Volunteering leads to rock-paper-scissors dynamics in a public goods game. Nature 425: 390–393. 14508487 10.1038/nature01986

[39] Skyrms B, Irvine RC (2001) The stag hunt. Proc. Address. Am. Phil. Assoc. 75: 31–41.

[40] Smith JM, Price JR (1973) The Logic of Animal Conflict. Nature 246: 15–18.

[41] Stander PE (1992) Cooperative hunting in lions: the role of the individual. Behav. Ecol. Sociobiol. 29: 445–454.

[42] Szabó G, Hauert C (2002) Phase transition and volunteering in spatial public goods games. Phys. Rev. Lett. 89: 118101. 10.1103/PhysRevLett.89.118101 12225171

[43] Taylor M, Ward H (1982) Chickens, whales, and lumpy goods: alternative models of public-goods provision. Politic. Stud. 30: 350–370.

[44] Traulsen A, Nowak MA (2006) Evolution of cooperation by multilevel selection. Proc. Natl. Acad. Sci. USA 103: 10952–10955. 10.1073/pnas.0602530103 PMC1544155 16829575

[45] Traulsen A, Hauert C, Silva HD, Nowak MA, Sigmund K (2009) Exploration dynamics in evolu-tionary games. Proc. Natl. Acad. Sci. USA 106: 709–712. 10.1073/pnas.0808450106 PMC2630064 19124771

[46] Perc M, Marhl M (2006) Evolutionary and dynamical coherence resonances in the pair approxi-mated prisoner's dilemma game. New Journal of Physics 8: 142.

[47] Chen X, Liu Y, Zhou Y, Wang L, Perc M (2012) Adaptive and bounded investment returns promote cooperation in spatial public goods games. PLoS ONE 7(5): e36895. 22615836 10.1371/journal.pone.0036895 PMC3353963

[48] Chen XJ, Szolnoki A, Perc M, Wang L (2012) Impact of generalized benefit functions on the evolution of cooperation in spatial public goods games with continuous strategies. Phys. Rev. E 85: 066133. 10.1103/PhysRevE.85.066133 23005188

[49] Trivers RL (1971) The evolution of reciprocal altruism. Q. Rev. Biol. 46: 35–57.

[50] Wakano JY, Nowak MA, Hauert C 2009 Spatial dynamics of ecological public goods. Proc. Natl. Acad. Sci. USA 106: 7910–7914. 10.1073/pnas.0812644106 PMC2683138 19416839

[51] Perc M, Wang Z (2010) Heterogeneous aspirations promote cooperation in the prisoner's dilemma game. PloS ONE 5(12): e15117. 21151898 10.1371/journal.pone.0015117 PMC2997779

